# Toxicological evaluation of environmental chemicals associated with AD identified using an exposomic framework

**DOI:** 10.1002/alz.087444

**Published:** 2025-01-09

**Authors:** Vrinda Kalia

**Affiliations:** ^1^ Mailman School of Public Health, Columbia University, New York, NY USA

## Abstract

Exposure to environmental chemicals has been associated with Alzheimer’s disease (AD); however, most studies have used a targeted approach to study this relationship. While targeted approaches have been critical to understand mechanisms, they do not reflect real world exposures where an individual is exposed to multiple chemicals at the same time. Exposomics provides the opportunity to use an ‐omics level approach to understand the environmental drivers of disease by measuring the burden of multiple chemicals at the same time. Using an exposomic approach, we found a metabolite of the legacy pesticide DDT to be associated with AD in an observational study. We then investigated whether DDT can exacerbate AD‐related pathology using a transgenic strain of the nematode model *Caenorhabditis elegans* (worm) that expresses a mutant tau protein fragment that is prone to aggregation.

Exposure to 3µM DDT resulted in internal levels of 0.5 pg (SD: 0.01) p, p’‐DDT per worm and 0.1 pg (SD: 0.01) of its metabolite p, p’‐DDE. We found that exposure DDT significantly restricted growth in the transgenic strain more than it does in wildtype worms. Further, DDT significantly lowered mitochondrial respiration rates in both strains. High‐resolution mass spectrometry‐based metabolomics (HRMS) analysis using the whole worm showed reduced levels of several amino acids and an increase in adenosylselenohomocysteine in DDT exposed worms. DDT exposure in the transgenic strain significantly increased the amount of time spent curling. The curling phenotype has been previously associated with mitochondrial dysfunction. Our data suggest that low level exposure to DDT likely exacerbates the mitochondrial inhibitory effects of aggregating tau protein in *C. elegans*.

We are investigating the relationship between persistent organic pollutants and age‐related cognitive decline in the Reference Ability Neural Network study. This study, conducted in healthy volunteers, allows us to consider the relationship between exposure and poor cognitive function without confounding by underlying disease. We plan to investigate the potential mechanism through which these chemicals affect neuronal health while considering them as a mixture using *C. elegans*. Recent advancements in predictive toxicology provide a starting point to consider the appropriate health and functional end points that should be investigated.

